# The Treatment Effect of Porous Titanium Alloy Rod on the Early Stage Talar Osteonecrosis of Sheep

**DOI:** 10.1371/journal.pone.0058459

**Published:** 2013-03-14

**Authors:** Xiao-Kang Li, Chao-Fan Yuan, Jun-Lin Wang, Yong-Quan Zhang, Zhi-Yong Zhang, Zheng Guo

**Affiliations:** 1 Department of Orthopaedics, Xijing Hospital, Fourth Military Medical University, Xi'an, China; 2 School of Stomatology, Fourth Military Medical University, Xi'an, China; 3 Department of Plastic and Reconstructive Surgery, Shanghai 9th People's Hospital, Shanghai Key Laboratory of Tissue Engineering, School of Medicine, Shanghai Jiao Tong University, Shanghai, China; 4 National Tissue Engineering Center of China, Shanghai, China; Ghent University, Belgium

## Abstract

Osteonecrosis of the talus (ONT) may severely affect the function of the ankle joint. Most orthopedists believe that ONT should be treated at an early stage, but a concise and effective surgical treatment is lacking. In this study, porous titanium alloy rods were prepared and implanted into the tali of sheep with early-stage ONT (IM group). The curative effect of the rods was compared to treatment by core decompression (DC group). No significant differences in bone reconstruction were observed between the two groups at 1 month after intervention. After 3 months, the macroscopic view of gross specimens of the IM group showed ordinary contours, but the specimens of the DC group showed obvious partial bone defects and cartilage degeneration. Quantitative analysis of the reconstructed trabeculae by micro-CT and histological study suggested that the curative effect of the IM group was superior to that of the DC group at 3 months after intervention. These favorable short-term results of the implantation of porous titanium alloy rods into the tali of sheep with early-stage ONT may provide insight into an innovative surgical treatment for ONT.

## Introduction

Osteonecrosis of the talus (ONT) may be caused by trauma, usage of prednisone, cytostatica treatment or some systematic immune diseases and so on [1]–[5]. Blockage or disturbance of the blood circulation is thought to be the direct cause of ONT. As a major load-bearing region, the talus provides mechanical support for the whole body weight of a human in the orthostatic state. The development of ONT may substantially alter the structural integrity of the talus and dramatically increase the intraosseous pressure of the talus, which may further disturb blood circulation through the talus [1]. Under the synergetic effects of the increase in intraosseous pressure and decrease in blood supply, without proper therapeutic intervention ONT may rapidly become aggravated and lead to eventual failure of the ankle joint.

Several therapeutic interventions are currently available for the treatment of ONT, including biophosphonate treatments, core decompression, vasotransplantation, and the transposition of vascularized bone flap. Biophosphonate could be used to treat the osteonecrosis, because of their capacity to inhabit the bone resorption. Core decompression and vasotransplantation are traditional approaches to the treatment of early-stage ONT that may help to reduce the intraosseous pressure and improve the blood circulation. However, they do not provide additional mechanical support, which would further reduce the intraosseous talar pressure [3]–[7]. These approaches also require long-term immobilization of the ankle joint during treatment, which could lead to osteoporosis due to a lack of mechanical stimulation [8]. Therefore, these approaches are associated with very limited success in clinical practice. The transposition of vascularized bone flap aims to treat ONT by reconstructing the bone tissue and providing vascularization. However, this method is associated with an uncertain long-term therapeutic outcome due to the poor survival rate of the implanted bone flap and a lack of sufficient mechanical support [9]–[11].

To address the decreased mechanical support of the inherent bone tissue associated with osteonecrosis, metal implants have been proposed as a treatment approach, especially for femoral head osteonecrosis [12]–[16]. Metal implants such as the porous tantalum rod (Trabeculaer Metal, Zimmer, USA) may provide additional mechanical support in the load-bearing region, which may help to maintain the structural integrity of the bone tissue, reduce the intraosseous pressure by creating the implantation canal, and improve blood perfusion for bone regeneration [12]–[13]. Tantalum rod implantation has been shown to achieve an 80% satisfactory clinical outcome for femoral head osteonecrosis treatment, especially in the early stage, with appropriate mechanical strength and little impingement of the stress shield [12]–[14]. Furthermore, metal rods can be implanted in a minimally invasive manner, thereby reducing damage to the surrounding tissues, and favoring the rapid regeneration of bone tissue in the later stage [13].

However, the use of metal implants has been limited to femoral head osteonecrosis treatment. To the best of our knowledge, there are no preclinical animal or clinical investigations of their potential use in treating ONT. Therefore, we hypothesized that the implantation of porous metal rods could be an effective surgical intervention for the treatment of ONT by providing sufficient mechanical support to reduce the intraosseous pressure and restore the blood supply, and by acting as a scaffold matrix to promote new bone regeneration. Recently, we have successfully established a preclinical sheep model of ONT with clinical relevance, which provides a good animal model for this hypothesis testing. By using the method of intraosseous injection of pure ethanol in the median talar head, we developed the animal model of ONT with clear sign of stage II osteonecrosis 4 weeks postoperatively, which is considered to be a proper time for surgical intervention [17]. Hence, in the present study, based on the development of this preclinical ONT animal model, we aim to investigate the efficacy of a porous titanium alloy rod, fabricated by the electron beam melting (EBM) technique, for ONT treatment.

## Materials and Methods

### Animals

All animal experiments were performed according to protocols approved by the Institutional Animal Care Committee of Fourth Military Medical University. Twelve adult female Small Tail Han Sheep (median age: 25 months, range: 20–31 months; median weight: 42.5 kg, range: 35–57 kg) were used in the study. The sheep were housed at the Department of Orthopedics of Xijing Hospital Animal Resources Center in two approved shacks with an average acreage of 18 m^2^. All sheep were maintained under routine husbandry.

### Fabrication of porous titanium rods using the EBM technique

Porous titanium rods were fabricated by three major procedures. First, the three-dimensional (3-D) rod structure was designed by computer-aided design (CAD). Data were saved in standard template library format and inputted into the EBM S12 system (Acram AB, Sweden). Second, titanium alloy powder (Ti_6_Al_4_V) was melted layer by layer in the Acram EBM S12 system and the rod structure was remolded according to the CAD model. Third, the residual powder was removed and the products were prepared.

**Figure 1:**
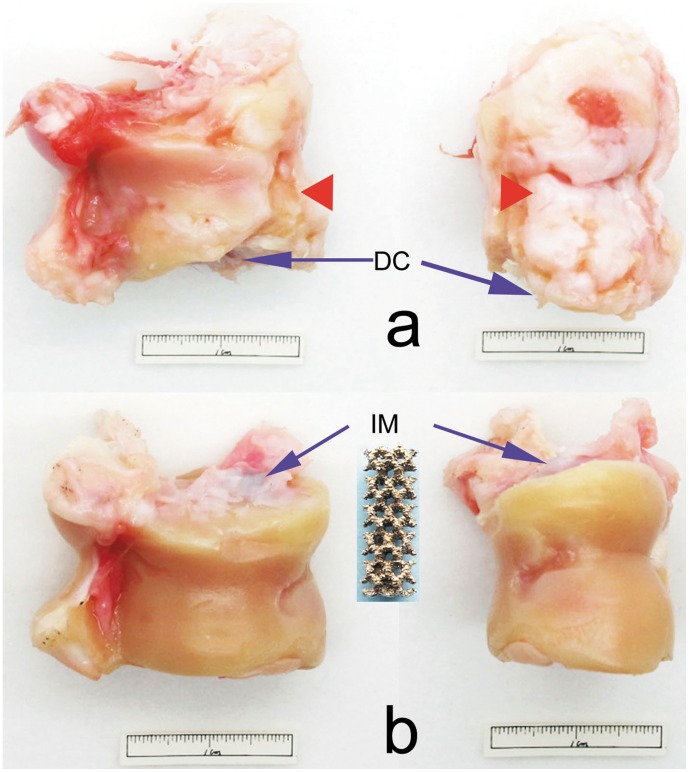
Sample of a porous titanium alloy rod and gross specimens at 3 months after treatment. Blue arrowheads indicate treatment sites.

To prepare the products, the titanium alloy powder was made into porous cylinders of 4 mm in diameter and 12 mm in length (IM, Figure 1). The porosity of the cylinders was 70% and the pore size was about 1 mm. The rods had an average compressive strength of 36.36 MPa, which is much higher than that of spongeous bone (Table 1). Based on these data, the elastic modulus of the porous rod as a unit was about 2.2 GPa, according to a previous study [18].

**Table 1:** Characteristics of the porous titanium alloy.

Sample mass, m (g)	Sample dimensions, (mm^3^)	Density, ρ(g/cm^3^)	Relative density, (ρ/ρ_s_)^a^	Porosity (%)	Compressive strength,σ (MPa)
0.45	φ4×12	1.33	0.3	70	36.36

### Experimental design

**Figure 2:**
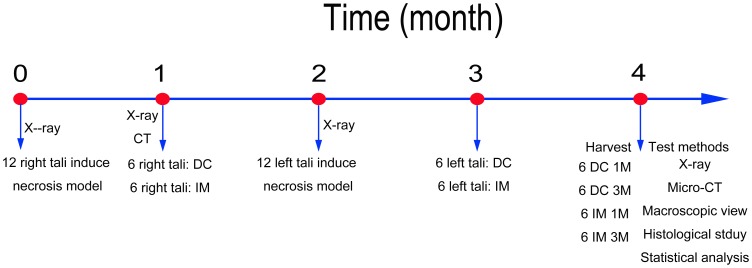
Schematic illustration of experimental design.

After the sheep were weighed, they were randomly and evenly divided into two groups. To induce ONT, each talus was administered an intraosseous injection of pure ethanol (Figure 2). At 1 month after ONT induction, the early stage of ONT was confirmed by radiographic and computed tomography (CT) examinations. Porous titanium alloy rods were implanted (group IM), and core decompression surgery was performed (group DC) on the necrotic tali. At 1 month after this intervention, ONT was induced in the contralateral tali of the sheep by the same method; rods were implanted, and core decompression was executed on these tali at 1 month after ONT induction. Therefore, six samples with different time points of each kind of treatment were harvested after 4 months.

The testing methods included macroscopic, radiographic, micro-CT, CT, and histological examinations. X-ray photographs were taken at 1 month after inducing the necrotic model, and at 1 and 3 months after the therapeutic treatments. One living sheep was randomly chosen for CT analysis at 1 month after ONT induction for the examination of talus necrosis. Micro-CT (threshold: 500–1400 HU) was performed to observe the 3-D structure inside the bone, with four random samples being taken at 1 and 3 months after intervention. The region of interest (ROI) was chosen as a cylinder (φ4.3×5 mm^3^) in the center of the core decompression drill track, which was considered to be the area with the most obvious bone necrosis.

### Surgical procedures

All surgical procedures were performed under sterile conditions and followed our previous experiences [17]. The sheep were fasted for 24 hours before the surgery, placed under general anesthesia, and fixed on an operating table in a lateral position. After the skin was shaved and disinfected with iodine solution, a straight incision of about 4 cm was made at the medial ankle. The fascia was separated, and the joint capsule was cut open until the center of the talar head was exposed. A hole (1.2 mm in diameter, 15 mm in depth) was drilled at the center of the medial talar head. Three milliliters of pure ethanol (Fuyu Corporation, Tianjin, China) were instilled into the talar head through the hole at a flow rate of 0.8 mL/min. To prevent liquid reflux, the hole was blocked with bone wax immediately after injection. The wound was then washed and closed in layers.

One month later, therapeutic treatments were performed on the tali in the early stage of necrosis. Under general anesthesia, the center of the medial talar head was exposed as described above, and a hole of approximately 4 mm in diameter and 13 mm in depth was drilled. For the DC group, the debris of the necrotic bone tissue was mostly removed. For the IM group, porous titanium alloy rods were implanted into the hole after removal of the necrotic bone tissue.

### Histological examinations

**Figure 3:**
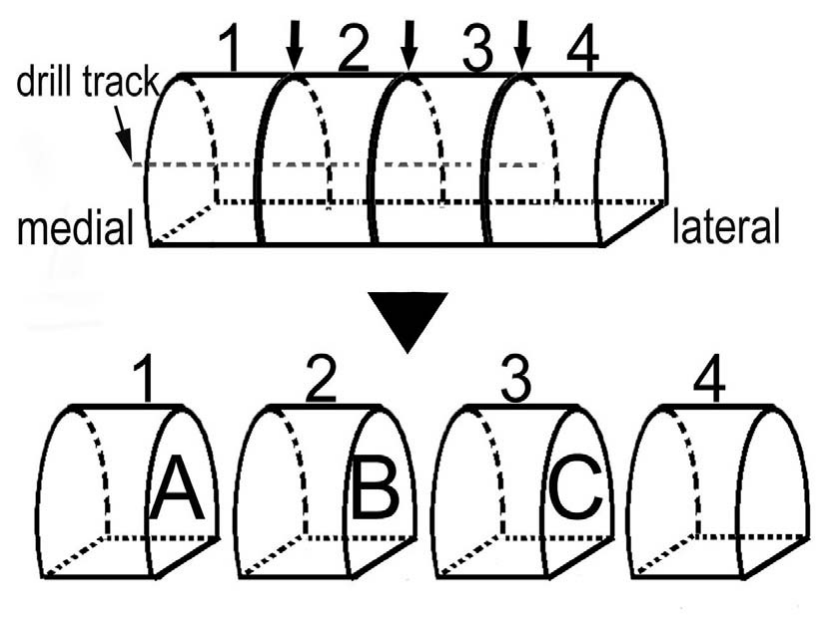
Section design for histology. Sections A, B, and C were prepared for staining.

The talar heads of all samples were cut off and fixed in 10% formalin for 2 weeks. Four specimens of each group at each time point were randomly chosen and scanned by micro-CT. All of the specimens were dehydrated for about 20 days and then embedded with synthetic resin. The individual specimens were evenly divided into four parts crossing the drill track, such that three different sections could be observed by histology (Figure 3). The sections (labeled A, B, and C) were cut to 150 µm thick and, after careful sanding, were stained with Van Gieson.

**Figure 4:**
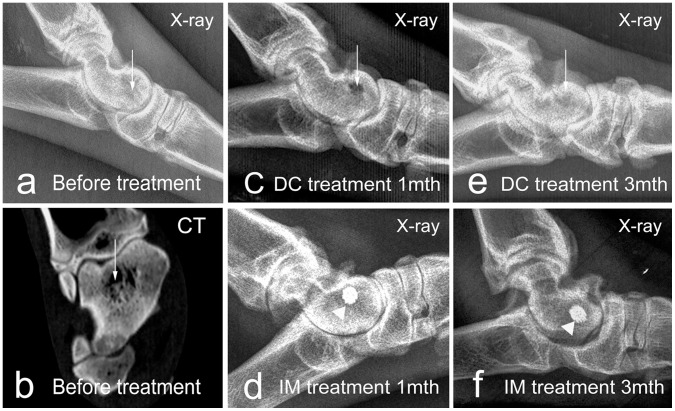
Histological examination of the DC and IM groups. The sections showed that the trabecular reconstruction was remarkable mainly in the IM group at 3 months after treatment, and the combination of the trabeculae and titanium alloy was very tight (Van Gieson stain, original magnification: left ×16; right ×100).

To evaluate and compare the extent of bone reconstruction, a new histological appraisal system was developed. A round ROI that just covered the hole of core decompression was made in the pictures of the tissue slices with a minimum magnification (black circle, magnification: ×16, Figure 4). The average percentage of the trabeculae in this area was calculated.

### Statistical analysis

Statistical analyses of samples at different time points (% of reconstructed trabeculae in the ROI) were performed by analysis of variance (ANOVA) with multiple comparisons. The SPSS 16.0 (International Business Machines Corporation, Armonk, New York, USA) program was used for all statistical analyses. Differences were considered significant at a value of *P<*0.05.

## Results

The sheep showed good physical conditions after surgery, with complete wound healing by 1 week later, without any complications of infection or pathological bone fracture. The sheep implanted with titanium alloy rods (IM group) regained the normal walking condition, except for a slight degree of lameness in the operated leg in the first 2 weeks post-operatively. The sheep treated by core decompression (DC group) went lame, showing an unwillingness to load the operated leg throughout the whole study.

### Macroscopic examination

At 1 month after the interventions (i.e., implantation of the titanium alloy rods and core decompression), the tali showed a normal appearance in all gross specimens. At 3 months after core decompression, tali in the DC group showed defects of about 5 mm×6 mm on the cartilage, and the stress-concentrated area of the talar heads had widely degenerated to an enlarged area (red triangle, Figure 1a). However, the tali of the IM group still maintained intact contours (Figure 1b).

### X-ray and CT examination

**Figure 5:**
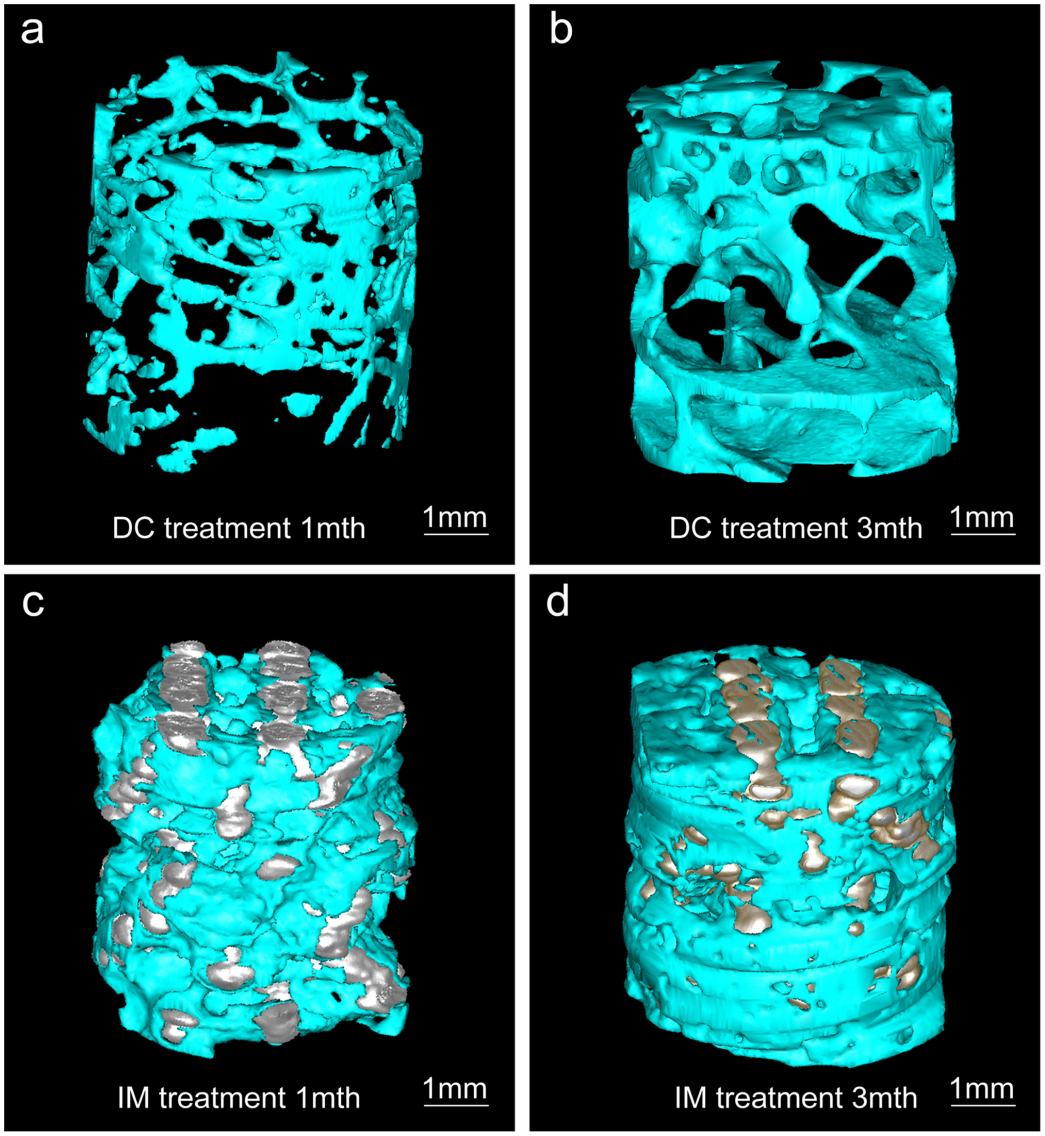
Lateral radiographic images of the ankle at different times. White arrowheads indicate osteolytic changes inside the talus, and white triangles indicate titanium rods.

At 1 month after the primary surgery to induce osteonecrosis, X-ray imaging showed an uneven radiographic density of the spongy bone tissue around the drill track, with the lowest density at the center of the talar head (white arrow head, Figure 5a). This finding was further confirmed by CT examination, which showed a necrotic core with a lower density (white arrow head, Figure 5b).

At 1 month after core decompression, the X-ray image of the DC group displayed an empty cavity (white arrow head, Figure 5c). At 3 months after intervention, this region was filled but possessed a lower X-ray density compared to the surrounding tissues (white arrow head, Figure 5e). By contrast, the IM group at 1 and 3 months post-intervention showed homogenous X-ray density of the surrounding bone tissue around the titanium alloy rod (white triangle, Figure 5d and f).

**Figure 6:**
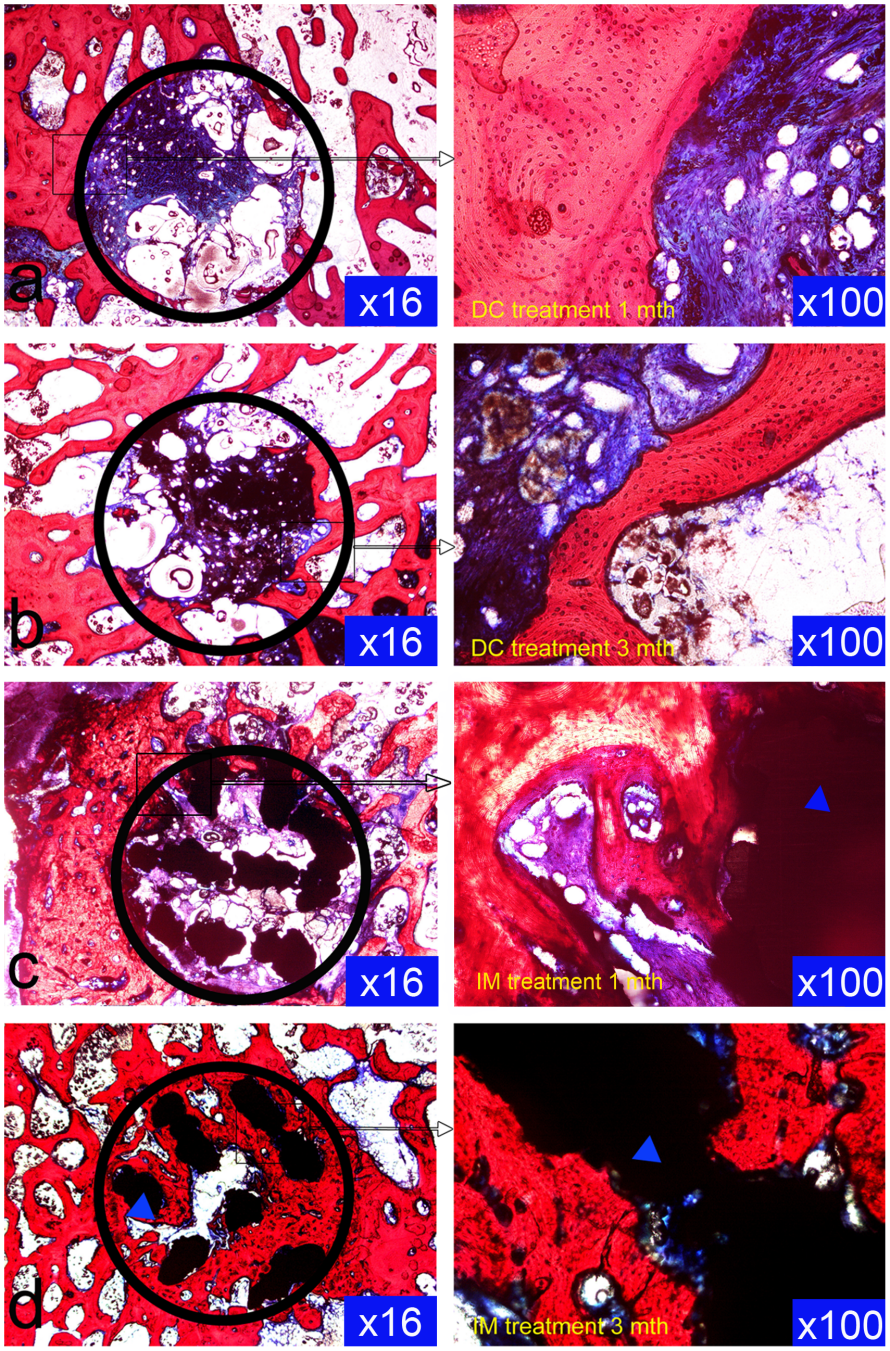
The 3-D reconstruction of talar specimens performed by micro-CT. Quantitative study showed that the trabecular reconstruction of the IM group was prior to that of the DC group at each time point.

Micro-CT, which was used to discover the visual and 3-D structures of the reconstructed trabeculae, revealed that the quantity and quality of reconstructed trabeculae in the IM group were better than those of the DC group (Figure 6). These findings were further confirmed by a quantitative analysis of the quantity and scale of the reconstructed trabeculae, through the trabeculae percentage and the trabeculae thickness and spacing, respectively (Table 2).

**Table 2:** Quantitative analysis of the parameters for reconstructed trabeculae, as calculated by micro-CT.

Time/Treatment	Trabeculae percentage	Trabeculae thickness (mm)	Trabeculae spacing (mm)
Month 1 DC	5%	0.08	1.59
Month 1 IM	20%	0.07	0.26
M**o**nth 3 DC	25%	0.18	0.52
Month 3 IM	37%	0.11	0.18

### Histological analysis

In the DC group, at 1 month after decompression treatment, the necrotic cavity was obvious, with little bone regeneration and moderate fibrous tissue infiltration (Figure 4a). At 3 months after treatment, the overall structure of the bone trabeculae inside the talus was loose and irregular, and was accompanied by more fibrous tissue (Figure 4b).

By contrast, the implantation of the titanium alloy rod (IM group) allowed much better bone regeneration. At 1 month post-treatment, we observed some fibrous tissue infiltration into the cavity area and a loose trabecular structure around the implanted titanium alloy rod. Nevertheless, bone tissue ingrowth was observed at the edge of the porous titanium alloy rod (blue triangle, Figure 4c), achieving tight integration of the titanium strut with the bone tissue. At 3 months after treatment, porous titanium alloy rod implantation led to robust bone regeneration in the cavity region, with copious bone tissue infiltration within the porous titanium alloy rod (blue triangle, Figure 4d) and the formation of a compact trabeculae structure of bone tissue surrounding the titanium implant. Almost no fibrous tissue was found inside the porous material.

### Quantitative analysis

Using the new histological appraisal system and statistical approach, we determined the percentages of reconstructed trabeculae in the ROIs of the two groups (Table 3). In terms of bone tissue regeneration, there was no difference between the DC and IM groups at 1 month post-treatment (*P>*0.05). At 3 months post-treatment, the IM group showed 1.7 times better bone regeneration than the DC group (*P*<0.05).

**Table 3:** Percentages of reconstructed trabeculae in the ROIs of the two groups by histological study (*n* = 6, x ± s).

Time	DC group	IM group	*P*
Month 1	0.058±0.015	0.078±0.020	0.482
Month 3	0.250±0.062	0.427±0.070	0.000

## Discussion

The clinicopathological symptoms of early-stage ONT include a continuous dull pain in the ankle region. The diagnosis may be further validated by magnetic resonance imaging. Late-stage ONT may lead to subchondral collapse of the talus, accompanied by pain and severe dysfunction of the ankle joint [19]. Therapeutic interventions at the early stage of ONT are recommended to prevent subchondral talar collapse, which otherwise may be treated by arthrodesis [2], [3], [20]. As an early therapeutic intervention strategy, biophosphonates treatment could be utilized, however, its efficacy may be controversial. For instance, Jureus et al. have reported an effectiveness ratio of 57% for the knee osteonecrosis treatment [21], while other studies showed that bisphosphonates treatments may further exacerbate the osteonecrosis through the potential side effect of anti-angiogenic [22].

Alternatively, surgical treatment could be utilized as well.

During the surgical treatment for osteonecrosis, the provision of additional mechanical support to prevent subchondral collapse is highly beneficial [23]. Moreover, Floerkemeier et al. recently suggested that bone reconstruction was possible if the subchondral collapse of the osteonecrotic femoral head had ceased at an early stage [15]. On the basis of these previous findings, we investigated the use of porous titanium alloy rods to treat ONT in a preclinical sheep model. We demonstrated that the porous titanium alloy rods not only provided strong mechanical support for the talus tissue but also worked as a scaffold matrix to promote bone regeneration in the necrotic region.

Several animal models have been established for the study of femoral head osteonecrosis. Methods to induce osteonecrosis include intraosseous injection of pure ethanol or cortical hormone, as well as intraosseous destructive treatment by microwave heating or using a cryogenic reagent (e.g., liquid nitrogen) [24]–[28]. Nevertheless, to date, no animal model for ONT has been developed before our research research work, which established a clinically relevant animal model to simulate ONT in humans [17]. Sheep were selected because of their similarity to humans in terms of anatomical structure and mechanical loading [29].

The ONT model established in our resent study imitated the pathological characteristics of human ONT in the following two aspects. First, the necrosis originated from the spongy bone area inside the talus and then gradually progressed to the region of cortical bone and cartilage. Second, the pathological progress of the induced ONT was necrobiotic, with obvious osteolytic appearance under radiographic examination at one month after induction [17]. This slower progression of necrosis is closer to the human ONT situation, compared to the rapid osteonecrosis induced by liquid nitrogen or microwave heating [25], [28]. Finally, this model preserved the necessary conditions for the subsequent surgical treatment because it did not completely destroy the talar blood circulation.

Using this preclinical ONT model, our study demonstrated that the implantation of a porous titanium alloy rod (IM group) achieved a better therapeutic outcome than the traditional core decompression treatment (DC group), especially in terms of bone tissue regeneration and reconstruction. The trabeculae of bone tissue at the necrotic region was much better restored in the IM group, with larger volume and a thicker and more interconnected structure, as evidenced by micro-CT and histological studies (Tables 2 and 3). By contrast, the trabecular structure in the DC group exhibited many ruptures and irregularities, indicating inferior bone reconstruction. However, the specimen of micro-CT was random chose and the artifact of the metal might influence the result, so we thought the histological data would be more credible. Despite of these positive findings in the current research work, a longer period of study with 6–12 months follow-up investigation may provide a better understanding of the long-term efficacy of this therapeutic strategy.

There are several possible explanations for the observed beneficial therapeutic effects of porous titanium alloy rod implantation. The porous titanium alloy rod provides sufficiently firm mechanical support for the surrounding bone tissue after implantation, which is able to maintain the integrity of the trabecular structure and reduce direct loading on the necrotic bone tissue, favoring bone tissue regeneration [30]. Due to the high elastic modulus of the Ti_6_Al_4_V material, the titanium alloy rod was fabricated in a porous structure with a porosity of 70%. As a result, the stress shield effect of the titanium alloy rod was greatly depressed [17]. The elastic moduli of the porous titanium alloy rod and trabecular bone were similar, which may enable long-term stability between these structures [31] and ensure the long-term therapeutic utility of this approach.

Moreover, titanium alloy is well known for its long-term in vivo biocompatibility [32]. The implanted porous titanium alloy rod may work as a scaffold matrix, thereby providing the framework with struts for osteogenic cell adhesion, proliferation, and extracellular matrix deposition, and promoting better and faster ingrowth of bone tissue in the necrotic region than the simple core decompression treatment in the DC group. This explanation was evidenced by the histological study, which showed the initial integration of bone tissue with the titanium alloy strut at 1 month, and the rapid infiltration of bone tissue at 3 months post-transplantation (Fig. 4).

In this study, EBM technology was used to fabricate the titanium implant with a precisely controlled porous structure for ONT treatment. EBM technology is widely used in the manufacturing of metal materials with intricate inner or surface structures, and could be used to produce any needed implant to replace a bone defect in orthopedics. As a rapid prototyping technique, EBM technology has many advantages compared to traditional sintering and laser beam fusion techniques, including: (1) its ability to prepare complex and irregular components; (2) its excellent repeatability, due to the use of a computer-controlled process; (3) its ability to manufacture products directly using raw material powder, which could be a high-strength and high-melting metal such as titanium alloy; and (4) its reduced consumption of time and energy [17], [33]–[36]. Therefore, it is both feasible and convenient to produce titanium alloy implants by EBM technology.

## Conclusion

The porous titanium alloy rods fabricated by EBM technology possess good characteristics in terms of mechanical properties and biocompatibility, and they show obvious advantages when used to treat early-stage ONT. Although the long-term therapeutic effects need to be tested further, the curative effects of the porous titanium alloy rods appear to arise from their role as a firm scaffold, and their provision of decompression.
